# Supplementary material for: Loss of MXRA8 Delays Mammary Tumor Development and Impairs Metastasis
Source: Int J Mol Sci. 2023 Sep 6;24(18):13730. doi: 10.3390/ijms241813730 (PMC10530983; doi:10.3390/ijms241813730)
Supplement: Supplementary file 1 [file ijms-24-13730-s001.zip › ijms-2564009-supplementary.pdf]

```
231MXRA8KO-O  100  CTGGGCCTGGGGCGCCGCTGTGGCTGGAGCCGTTGCCCGGAGAGCCCCgggggggCGGCT  159
MXRA8-201     1000 CTGGGCCTGGGGCGCCGCTGTGGCTGGAGCCGTTGCCCGGAGAGCCCCGGGGGGGCGGCT  941

231MXRA8KO-O  160  CCGCGTGGGGTTCGGCGACCGTCAGGTGGAAGACGCGGCGTTCGTGCAGGCCACAGTAAT  219
MXRA8-201     940  CCGCGTGGGGTTCGGCGACCGTCAGGTGGAAGACGCGGCGTTCGTGCAGGCCACAGTAAT  881

231MXRA8KO-O  220  GGTGGTGCAGGTGGCAGGAGTAGGTGCCC------------------GGCTCGATACGCA  261
MXRA8-201     880  GGTGGTGCAGGTGGCAGGAGTAGGTGCCCTCGTCGGCGACCTCCAGCGGCTCGATACGCA  821

231MXRA8KO-O  262  GTGAGAAGTCACCGCGCTCAAAGGCATCCGCGCCCACAGCCACGCGGTCGCGCAGAAAAA  321
MXRA8-201     820  GTGAGAAGTCACCGCGCTCAAAGGCATCCGCGCCCACAGCCACGCGGTCGCGCAGAAAAA  761


231MXRA8KO-V  102  CTGGGCCTGGGGCGCCGCTGTGGCTGGAGCCGTTGCCCGGAGAGCCCCGG----------  161
MXRA8-201     1000 CTGGGCCTGGGGCGCCGCTGTGGCTGGAGCCGTTGCCCGGAGAGCCCCGGGGGGGCGGCT  941

231MXRA8KO-V  162  ------------------------------------------------------------  221
MXRA8-201     940  CCGCGTGGGGTTCGGCGACCGTCAGGTGGAAGACGCGGCGTTCGTGCAGGCCACAGTAAT  881

231MXRA8KO-V  223  ---------------------------------CGGCGACCTCCAGCGGCTCGATACGCA  263
MXRA8-201     880  GGTGGTGCAGGTGGCAGGAGTAGGTGCCCTCGTCGGCGACCTCCAGCGGCTCGATACGCA  821

231MXRA8KO-V  264  GTGAGAAGTCACCGCGCTCAAAGGCATCCGCGCCCACAGCCACGCGGTCGCGCAGAAAAA  323
MXRA8-201     820  GTGAGAAGTCACCGCGCTCAAAGGCATCCGCGCCCACAGCCACGCGGTCGCGCAGAAAAA  761
```

Figure S1. Sequencing results from 231MXRA8KO-O and 231MXRA8KO-V cells aligned to MXRA8-201 sequence

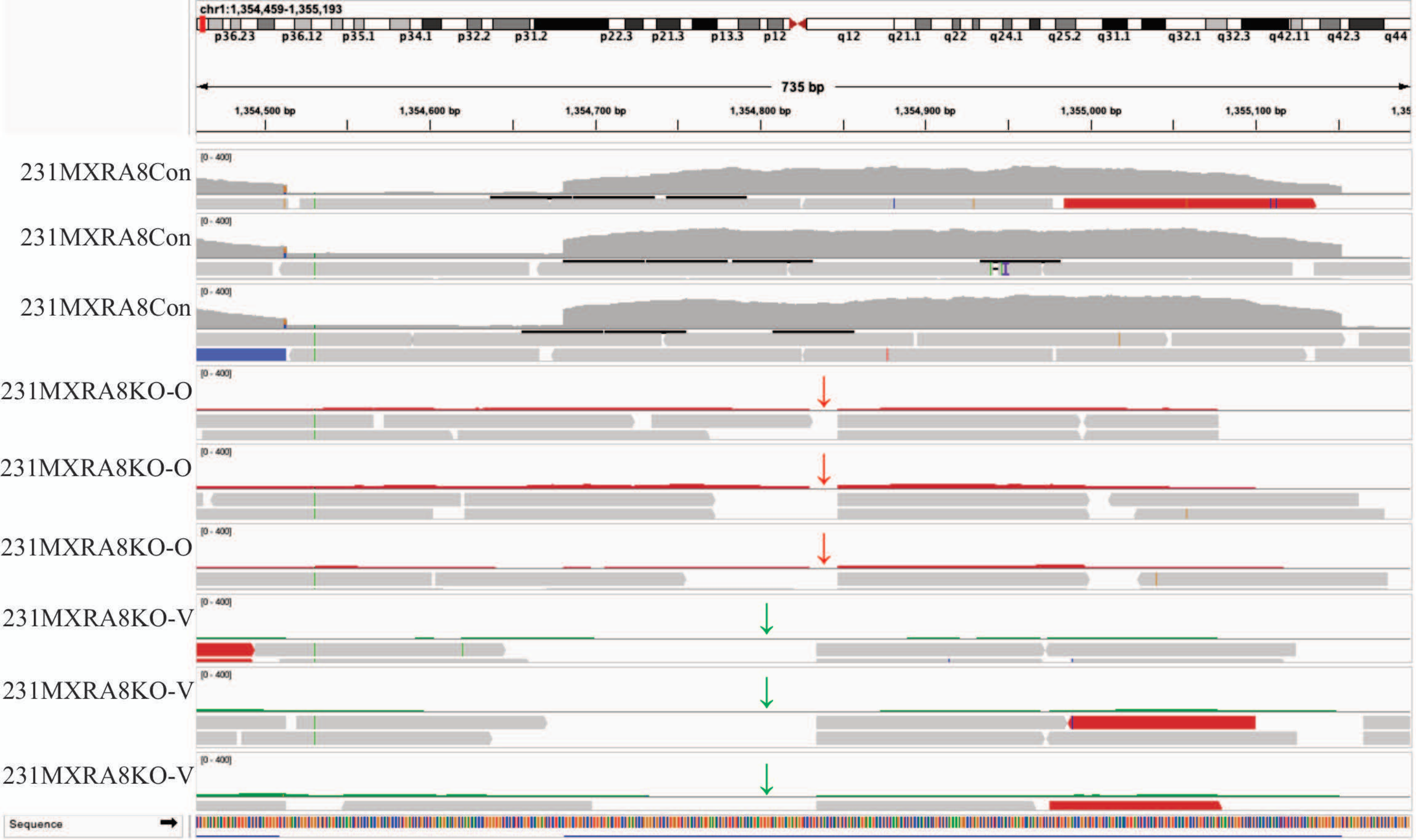

Figure S2. IGV image showing mapped reads from RNA sequencing data of 231MXRA8Con, 231MXRA8KO-O, and 231MXRA8KO-V cells grown in 2D culture. The red arrows indicate missing reads in 231MXRA8KO-O cells while the green arrows indicated missing reads in 231MXRA8KO-V cells.

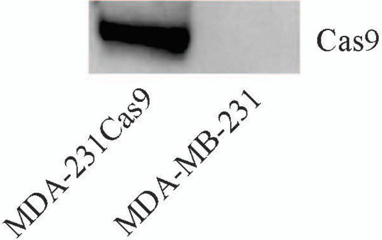

Figure S3. Western blot showing Cas9 protein in MDA-231Cas9 cells compared to parental MDA-MB-231 cells
